# Profitable third-party punishment destabilizes cooperation

**DOI:** 10.1073/pnas.2508479122

**Published:** 2025-08-19

**Authors:** Raihan Alam, Tage S. Rai

**Affiliations:** ^a^Rady School of Management, University of California, San Diego, CA 92093

**Keywords:** cooperation, punishment, morality, signaling, culture

## Abstract

Third-party punishment is theorized by some scholars to be essential to the evolution of large-scale cooperation, but empirically, it often fails to bring about its desired effects. Here, we suggest that third-party punishment destabilizes cooperation when third parties have profit motives to punish. Across nine economic games and judgment experiments (including four preregistered studies), we find that when third-party punishment is profitable, rates of cooperation decrease immediately and remain lower even when punishment outcomes are optimized to support cooperative behavior. Profitable third-party punishment causes targets of punishment to anticipate antisocial punishment and perceive social norms in terms of self-interest, suggesting that the introduction of payment degrades the communicative signals that punishment is meant to convey about punishers’ intentions and social norms. Critically, participants who would benefit from increased cooperation inadvertently reduce their own monetary compensation by opting in to experimental conditions that pay punishers, suggesting that they intuitively fail to consider the signaling consequences of profit motives to punish. Implications for systems of punishment and cooperation in real-world contexts are discussed.

## Significance

Third-party punishment is theorized by some scholars to be necessary for large-scale cooperation in social groups. Yet, punitive policies often fail to deliver cooperative benefits in real-world contexts. Here, we argue that people may have material incentives to punish that degrade its communicative signals about punishers’ motives and social norms. We find that paying third parties to punish destabilizes cooperation by causing targets of punishment to contribute less to others. Social groups are unaware of these dynamics and respond by materially incentivizing severe punishment that erodes cooperation and destroys their own welfare. These findings raise questions about the role of punishment in the evolution of cooperation and suggest that policymakers should consider alternative approaches to reducing crime.

Third-party punishment, which occurs when people who are not directly victimized themselves impose material costs on selfish actors, is theorized by some scholars to be fundamental to the evolution of large-scale cooperation in human societies (1–7). However, other scholars have questioned this relationship, noting that the costs of enacting punishment often exceed the cooperative gains it produces (8–12), and criminalized behaviors often persist despite severe punishment (13–16). The relationship between punishment and cooperation is also weak in societies characterized by low trust and corrupt institutions (17–19). Meanwhile, studies of punishment suggest that people care deeply about a punisher’s intentions, prefer punishments with clear intentions to punishments that are severe, and use a punisher’s intentions to infer morally correct behavior (20–28). Combining these strands of research, we argue that it may not be the imposition of material costs per se that is crucial for punishment to foster cooperation, but rather it is the information that punishment signals about punishers’ intentions and social norms that matter most. Social groups that are insensitive to these communicative signals may continue to use punishment to increase cooperation even under conditions where it is ineffective.

In this paper, we directly explore the communicative signaling function of punishment in cooperation by paying third parties to punish selfish behavior. A simple rational actor model suggests that paying third parties to punish should increase their willingness to do so, which in turn should increase rates of cooperation. However, we hypothesize that the introduction of monetary payment to punish degrades the communicative signal that punishment conveys about a punisher’s intentions and the social norms that apply in the situation. The target of punishment can no longer be certain whether punishers’ actions are due to moralistic or profit-seeking motives, and so they cannot reliably predict that punishers will refrain from harming them even if they cooperate, or that cooperation is even normative in this context (20–28). As a result, paying third parties to punish will break down rather than facilitate cooperation regardless of the effect that payment has on punitive behavior. We further hypothesize that people are metacognitively unaware of these signal-degrading effects of payment, and so they will choose to materially incentivize third-party punishment even as it fails to promote increased rates of cooperation.

## Overview of Studies

We investigate our hypothesis in a series of economic game and judgment experiments. In standard third-party punishment games, two players (dictator and receiver) are allocated a sum of money, but the dictator has complete control over how the money will be allocated between the two players. After observing the dictator’s allocation decision, a third player (punisher) can pay a monetary cost from their initial endowment to eliminate money from the dictator. The eliminated money is not reallocated to the punisher or the receiver. Such “costly punishment” allows experimenters to draw stronger inferences about punishers’ genuine preferences for prosocial punishment rather than self-interest. We created a profitable third-party punishment game that inverts this experimental design choice by paying punishers a monetary bonus to punish so as to obscure their motives to other participants (26–28). Prior studies using similar approaches have focused either on the effects of profit motives on punisher behavior (26) or inferences of truthful information (28), rather than our focus on downstream cooperative sharing of resources by dictators. Participants in all of our economic game experiments were informed that their decisions were binding and would be entered into lotteries to be played out for real monetary stakes.

Experiment 1 employs a one-shot game that varies the presence of a bonus payment to punish to demonstrate that the introduction of profitable punishment reduces the dictator’s initial willingness to cooperate prior to any punishment exposure. Experiment 2 uses a repeated games design to show that the effect of profitable punishment on reducing dictators’ willingness to cooperate persists across rounds following punishment exposure. Experiment 3 finds that even under optimal learning conditions in which selfish behavior is always punished and sharing is never punished, cooperation rates in profitable punishment conditions improve but never recover. Experiment 4 finds that cooperation can be restored under profitable punishment by explicitly altering the structure of the game to signal that punishers cannot engage in antisocial punishment. In Experiment 5, we conduct a preregistered internal meta-analysis across Experiments 1 to 4 to show that the destabilizing effect of profitable punishment on cooperation is robust. In a preregistered experiment (Experiment 6), we allow receivers to choose the condition they are assigned to and instruct them to make choices that will maximize their individual earnings. We find that receivers are significantly more likely to choose to play with paid punishers who can inflict severe punishment, reducing their own monetary payouts in the game by about 20% compared to if they had not chosen to play with a punisher at all. Experiments 7 to 9 (including two preregistered experiments) use attitudinal judgment methods to explore meta-cognitive beliefs about profitable punishment that drive these effects.

## Experiment 1: Profitable Third-Party Punishment Destabilizes Cooperation

Experiment 1 (N = 950) employed a one-shot profitable punishment economic game using a between-subjects design. In the profitable punishment game, the dictator (Player 1) receives $20 and must make a dichotomous choice about whether to keep the entire allocation or split it equally with a receiver (Player 2). The punisher (Player 3) must decide whether to eliminate money from the dictator’s allocation. The receiver cannot alter the dictator’s allocation decision. Following standard protocol for economic game experiments, we do not label players as “dictators” or “receivers” or “punishers” nor do we use normative terms such as “punish” to minimize demand effects.

Dictators were randomly assigned to one of five conditions. In the control condition, no punisher was present. In the two unpaid punisher conditions, dictators were informed that a punisher could reduce either $5 (low punishment severity condition) or $10 (high punishment severity condition) from their final amount regardless of their allocation decision. The two paid punisher conditions were identical except that dictators were informed that punishers would receive an unspecified monetary bonus for punishing.

**Fig. 1.**
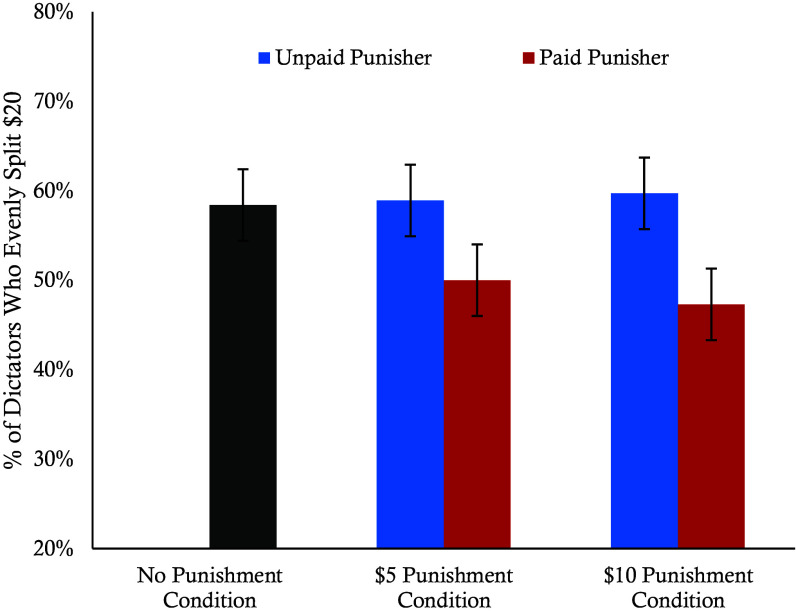
Cooperation rates between conditions. This graph shows the percentage of participants who evenly split the $20 with Player 2 for each condition from Experiment 1. Error bars represent SE.

We examined cooperation rates across the paid, unpaid, and no punisher control conditions using a binary logistic regression, with the no punisher control condition as the reference group. Results revealed that the introduction of profitable punishment significantly reduced dictators’ willingness to cooperate compared to the no punisher control condition (β = −0.39, *P* = 0.03), while cooperation in the unpaid punisher condition did not differ from the control condition (*P* = 0.89). We ran a second model that only included the four punishment conditions and added punishment severity as a covariate. The negative effect of profitable punishment on cooperation remained significant (β = −0.43, *P* = 0.003), while punishment severity had no main effect (*P* = 0.79). In Fig. 1 we plot the raw cooperation rates across the five conditions.

These findings indicate that the introduction of profitable punishment reduces initial cooperative tendencies prior to punishment exposure. In the next two experiments, we examine how the introduction of profitable punishment affects rates of cooperation prior to and following punishment exposure.

## Experiment 2: Effects Persist Following Punishment Feedback

Experiment 2 employed a repeated round profitable punishment economic game using a within-subjects design. Across rounds, punishers could remove money from dictators either with or without receiving a monetary bonus payment for doing so. Dictators experienced no punisher, paid punisher, and unpaid punisher conditions, allowing us to test how cooperation evolves in response to changes in punisher incentives both before and after exposure to punishment. If dictators respond to punishment using a simple rational model, then rates of cooperation should increase over time in paid punisher conditions if prosocial punishment increases more than antisocial punishment. Alternatively, if the communicative signal of punishment is degraded when punishment is profitable, then rates of cooperation may decrease in paid punisher conditions prior to any experience of punishment and may persist regardless of actual punishment patterns.

To test our hypothesis, we conducted a 12-round, simultaneous-player repeated game (N = 668) using SMARTRIQS (29). For the first four rounds, dictators played without any punishers, while punishers answered filler questions. For the next eight rounds, we introduced punishers. Half of the dictators played with paid punishers in rounds 5 to 8 and then unpaid punishers in rounds 9 to 12, while the other half of the dictators experienced the reverse order. In rounds 5 to 12, we also varied punishment severity such that punishers could remove $5 or $10 from dictators. Participants were informed that they would never play with the same partner in consecutive rounds. In the paid punishment rounds, punishers received a 5-cent bonus every time they removed money from dictators following their allocation decision. Dictators knew when they were playing with paid and unpaid punishers, but the amount of the bonus payment was unspecified to them.

**Fig. 2.**
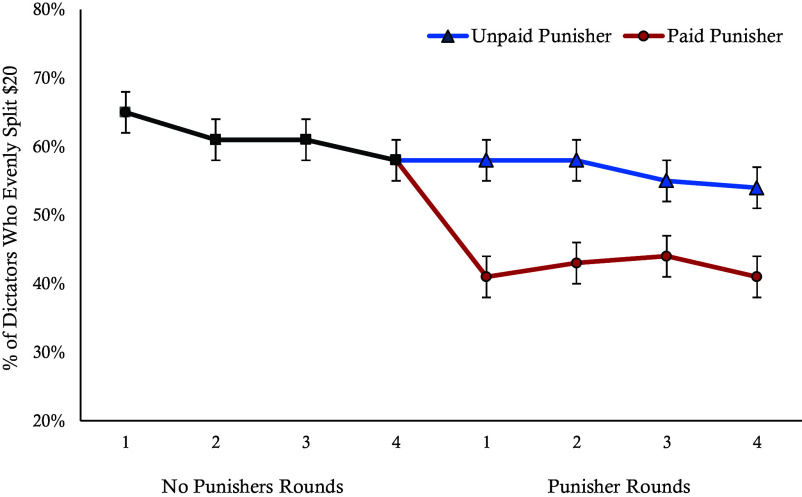
Cooperation rates between payment conditions across rounds. This graph shows the percentage of participants who evenly split the $20 with Player 2 per round for each condition from Experiment 2. Error bars represent SE.

We examined whether cooperation rates were influenced by switching between paid and unpaid punishers across three blocks of rounds: 1 to 4 (no punishers), 5 to 8 (paid and unpaid punishers), and 9 to 12 (conditions switched). For our primary analyses, we collapsed across levels of punishment severity and round order (i.e., whether participants encountered paid or unpaid punishers first) as they do not affect the main results (see *SI Appendix* for analyses examining interactions with punishment severity and payment condition order). To account for the repeated measures structure, where all participants experienced both paid and unpaid punisher conditions, we used a generalized linear mixed-effects model with participant included as a random effect. First, we analyzed cooperation rates in rounds 1 to 4 and found that rates of cooperation dropped across rounds (β = −0.82, *P* < 0.001), showing that, as in other studies, cooperation decreases in the absence of punishment (4–5). We next examined the effects of introducing punishment by collapsing the unpaid and paid punisher conditions across relevant rounds (5 to 8 and 9 to 12) from each sequence order. We find that unpaid punishers stabilize cooperation, with no significant differences in rates of cooperation compared to the last round without a punisher (β = −0.12, *P* = 0.54). In contrast, rates of cooperation drop significantly in the presence of paid punishers (β = −1.81, *P* < 0.001). In Fig. 2 we plot the raw cooperation percentages across incentive conditions.

Next, we examined how the introduction of payment affected punishers’ willingness to engage in prosocial and antisocial punishment. We used a generalized linear mixed-effects model with participant included as a random effect. We found that when dictators kept all of the money, paid punishers engaged in prosocial punishment significantly more (72%) than unpaid punishers (51%; β = 1.14, *P* < 0.001). They also engaged in antisocial punishment significantly more (23%) than unpaid punishers (8%; β = 1.84, *P* < 0.001) when dictators split the money equally. However, reduced cooperation in paid punisher conditions does not appear to be a response to increased antisocial punishment, as decreases in cooperation occur in the first round with paid punishers prior to any experience of punishment and remain stable following experiences of punishment across rounds. Further, rates of prosocial punishment increase marginally more than rates of antisocial punishment when punishers are paid (β = −0.43, *P* = 0.065), suggesting that the benefits of increased prosocial punishment following the introduction of payment to punish should have been equal to or larger than any moral hazard costs of increased antisocial punishment.

## Experiment 3: Optimal Reinforcement Learning Cannot Repair Signaling Costs of Profitable Third-Party Punishment

While we find little evidence in Experiment 2 to suggest that reduced cooperation between conditions is due to moral hazard costs, we investigate this question directly in Experiment 3 by providing identical feedback to dictators that is optimized for learning from punishment. Participants (N = 994) in a between-subjects design played the profitable punishment game as dictators for $20 and were informed they were playing with either a paid or unpaid punisher depending on condition who could remove $10 from them in each round for eight rounds, equivalent to the high punishment condition from Experiments 1 and 2. However, the experiment was programmed so that participants were always prosocially punished if they kept all of the money and never antisocially punished for sharing the money with receivers. As a result, regardless of the decision that dictators made, they would receive $10 every round, with the only difference being whether receivers would receive any money. This is the only economic game experiment that uses deception, which was necessary to maximize the causal effects of reinforcement learning. Importantly, optimized feedback is not uncommon; approximately 30% of punishers in Experiment 2 fit the profile we programmed in Experiment 3. The between-subjects design also allows participants to play eight rounds instead of four with one kind of punisher, increasing their opportunity to learn about punishment patterns in their assigned condition. If punishment sustains cooperation through reinforcement learning, then rates of cooperation should recover from their initial decline and match levels in unpaid punishment conditions under these conditions. At the very least, any initial gap in rates of cooperation between conditions should start to close. However, if the presence of profit motives degrades the communicative signal of punishment, then the gap in rates of cooperation may persist even as participants receive optimal punishment feedback for supporting cooperation.

**Fig. 3.**
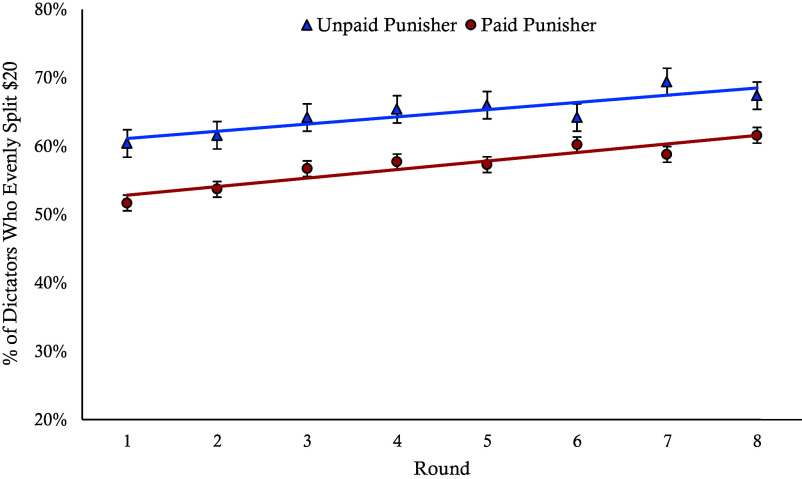
Cooperation rates between payment conditions across rounds. This graph shows the percentage of participants who evenly split the $20 with Player 2 per round for each condition from Experiment 3, when learning was optimized. Lines are regression lines, and error bars represent SE.

We analyzed cooperation rates across eight rounds using a generalized linear mixed-effects model with participant as a random effect to account for repeated measures. We found a significant effect of condition (β = 1.28, *P* < 0.001) and a significant effect of round number (β = 0.15, *P* < 0.001). As can be seen in Fig. 3, cooperation is higher across rounds in the unpaid punisher condition compared to the paid punisher condition, and cooperation increases across rounds in both conditions. However, the interaction between round and condition was nonsignificant (*P* = 0.53), such that there were no signs of convergence in cooperation rates over time even as participants received optimal punishment feedback. Thus, while learning occurs in the paid punisher conditions when punishment feedback is programmed to be perfect, the initial signaling costs from paid punishment persist as the gap in cooperation rates between conditions never closes across 8 rounds.

## Experiment 4: Explicitly Signaling That Punishers Cannot Antisocially Punish Eliminates Destructive Effects of Profitable Third-Party Punishment

The findings from Experiment 3 suggests that reinforcement learning through punishment is an inefficient means to repair the signaling costs of profitable punishment on cooperation even when it is optimized for learning. In Experiment 4, we investigate whether we can reduce the signaling cost of profitable punishment by altering the structure of the game so that punishers may only eliminate money from dictators who keep the entire allocation. This design choice allows us to examine whether cooperation in the paid punisher condition can be restored when dictators no longer have to worry about the potential for antisocial punishment of fair offers and when game instructions signal that the purpose of punishment is to reduce selfish behavior by dictators.

Participants (N = 1,011) were assigned to one of four conditions as dictators in a one-shot game in which they were either paired with an unpaid or a paid punisher, and in which they were either told that the punisher could punish them regardless of their allocation decision or that the punisher could only punish them if they kept all of the money. In all conditions, participants were told punishers could reduce their final payoff by $10.

**Fig. 4.**
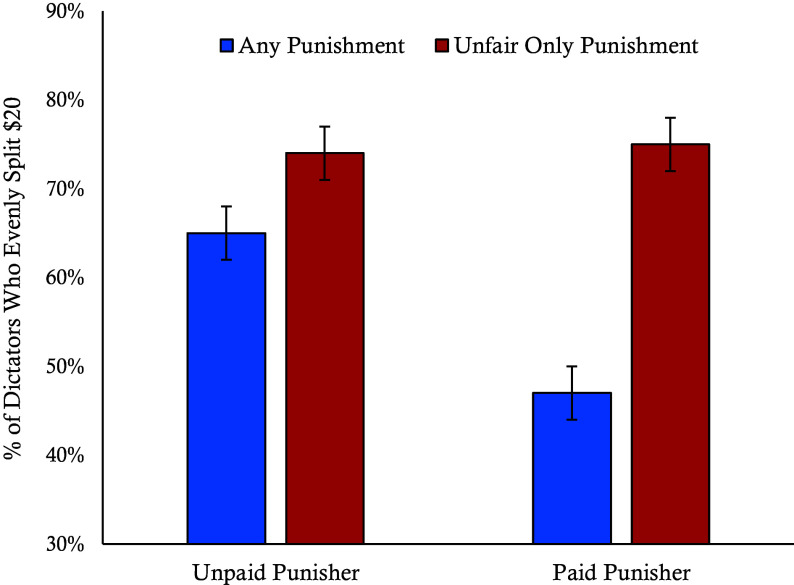
Interaction between payment and punishment structure conditions on cooperation rates. This graph shows the percentage of participants who evenly split the $20 with Player 2 depending on whether they were playing with paid or unpaid punishers and whether antisocial punishment was possible from Experiment 4. Error bars represent SE.

We conducted a binary logistic regression to examine the effects of game structure and incentives on cooperation. The results revealed significant main effects for both, such that participants were more likely to cooperate when punishers were unpaid compared to when they were paid (β = 0.72, *P* < 0.001), replicating the pattern found between paid and unpaid punisher conditions in Experiment 1, and they were more likely to cooperate when antisocial punishment of fair offers was impossible compared to when antisocial punishment was possible (β = 1.18, *P* < 0.001). Critically, there was a significant interaction (β = −0.76, *P* = 0.005), such that eliminating the potential for antisocial punishment had a stronger effect when punishers were paid (Fig. 4). The pattern of these results suggests that restructuring the rules of the game so that punishers can only punish unfair offers improves cooperation by eliminating dictators’ concerns about the potential for antisocial punishment and more strongly signaling cooperative social norms in both conditions. However, these effects are pronounced when punishers are paid, suggesting that the introduction of profit motives degrades the communicative signals that third-party punishment otherwise provides about punishers’ intentions and social norms.

## Experiment 5: Internal Meta-Analysis of the Effect of Profitable Third-Party Punishment On Cooperation

**Fig. 5.**
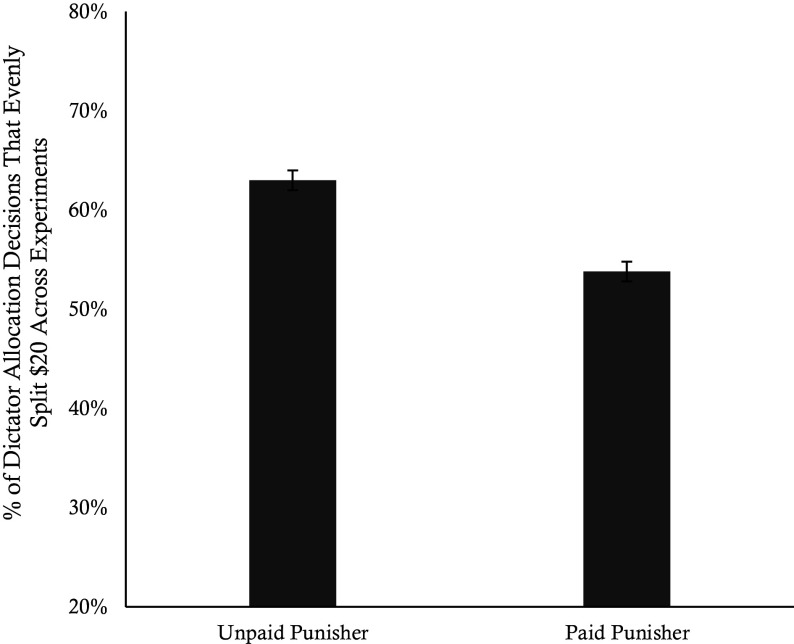
Cooperation by incentive conditions across experiments. This graph shows the percentage of participants who evenly split the $20 for each incentive condition from Experiments 1 to 4. Error bars represent SE.

To examine whether the effect of profitable punishment on cooperation is robust across study designs, we conducted a preregistered internal meta-analysis using data from Experiments 1 to 4. This yielded a combined sample of 3,099 participants who played as dictators and made 12,395 allocation decisions, given the repeated measures from Experiments 2 and 3. We fit a generalized linear mixed-effects model predicting cooperation as a function of punishment condition (paid vs. unpaid), with random intercepts for participant, round number, and experiment. The model revealed a significant effect of condition, such that participants were significantly less likely to cooperate when playing with a paid punisher (β = −1.62, *SE* = 0.13, *z* = −12.92, *P* < 0.001). Cooperation rates in the paid conditions are overestimated, as they include conditions with optimal feedback (Experiment 3) and an altered game structure to prevent antisocial punishment (Experiment 4) that partially mitigate the signaling costs of profitable punishment. See Fig. 5 for raw cooperation percentages by condition.

## Experiment 6: Receivers Choose to Play in Profitable Third-Party Punishment Conditions

Our experiments show that paying third parties to punish can reduce cooperation. In Experiment 6, which was preregistered, we examined participants’ metacognitive awareness of these punishment dynamics and whether they make suboptimal choices by allowing them to choose what conditions they will play the game in as receivers (Player 2). Receivers were participants (N = 399) who were recruited to be paired with dictators and punishers in Experiment 1 if they were selected for a lottery to have their game played out. Receivers were instructed that they would be able to choose whether they wanted to play with a punisher at all and what kind of punisher they would be assigned to play with. They were instructed that their goal was to make choices that would increase dictators’ willingness to share the allocation to maximize the amount of money they would make as receivers. Participants were informed that punisher conditions varied in whether the punisher is paid to punish and in the severity of punishment that is applied.

A binomial test revealed that most participants chose to play with a punisher (67%; *P* < 0.001), and a chi-square goodness-of-fit test confirmed that the overall distribution of choices significantly deviated from equal preference across conditions, χ²(2) = 20.63, *P* < 0.001. A plurality of participants (42.5%) chose to play with a paid punisher, more than those who selected an unpaid punisher (24.5%) or no punisher (33%). Among participants who decided to play with a punisher (N = 268), most chose to play in conditions where punishers were paid (64%; *P* < 0.001) and where punishment was severe (59%; *P* = 0.003). Finally, we examined preferences for severity among the participants who chose to pay punishers (N = 171) and found that most participants who decided to pay punishers chose more severe punishment (61%; *P* = 0.004).

**Fig. 6.**
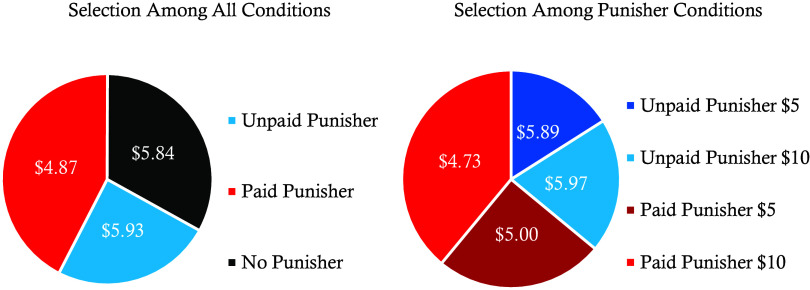
Player 2 expected values by chosen conditions. This graph shows the amount of money participants are expected to earn based on the conditions they chose as receivers from Experiment 6.

We can use dictators’ cooperation rates across conditions in Experiment 1 to calculate the expected payoffs of receivers’ decisions to evaluate the optimality of their choices. As shown in Fig. 6, receivers who choose to play in the control condition with no punishers expect to make $5.84 on average, which is not significantly different from the minority of receivers who choose to play with unpaid punishers and expect to make $5.93, χ^2^(1, 566) = 0.04, *P* = 0.86, but is significantly greater than the majority of receivers who choose to play with a paid punisher and expect to make $4.87, χ^2^(1, 574) = 4.81, *P* = 0.03, φ = 0.09. Among receivers who choose to play in conditions with punishers, the majority of them choose to play with paid punishers who engage in severe punishment, yielding the lowest expected payout of $4.73, a net loss of almost 20% in earnings compared to playing with no punisher at all.

## Experiments 7 to 9: Attitudes Toward Profitable Punishment

Our results thus far indicate that profitable punishment destabilizes cooperation, but that when given the choice, people choose to play with paid punishers, even as it destroys their earnings. We next conducted a series of judgment experiments to more directly assess participants’ attitudes and expectations of other players’ game behavior and social norms when punishment is profitable, and how different roles affect players’ perspectives to understand the causal mechanisms driving these dynamics.

**Fig. 7.**
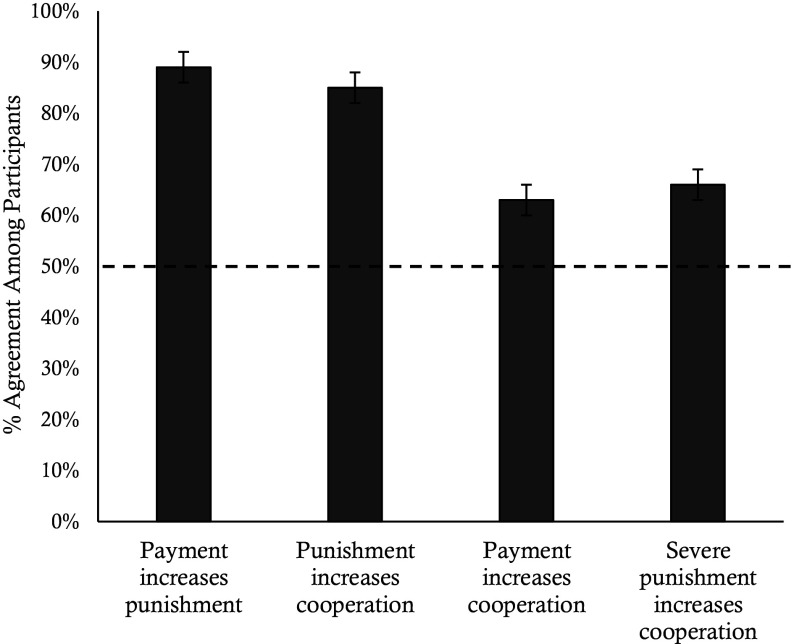
Naïve participant meta-beliefs about incentives and punishment. The graph shows participants’ expectations about the game based on agreement with the stated propositions from Experiment 7. The dashed line represents chance (50%), and error bars represent SE.

In Experiment 7, we explained how the game worked to naïve participants (N = 286) and asked them a series of questions about players’ likely behavior. 85% of participants believed that dictators would be more likely to share money with receivers if a punisher was present (*P* < 0.001) and 89% of participants believed that the punisher would be more likely to eliminate money from dictators if they received a monetary bonus payment for doing so (*P* < 0.001). Consequently, most participants (63%) believed that receivers would receive more money if they played with a paid punisher (*P* < 0.001). Additionally, we found that 66% of participants believed that more severe punishment would increase cooperation (*P* < 0.001). These results suggest that participants in Experiment 6 selected into profitable, severe punishment conditions because they falsely believed that such conditions would maximize their earnings (Fig. 7).

**Fig. 8.**
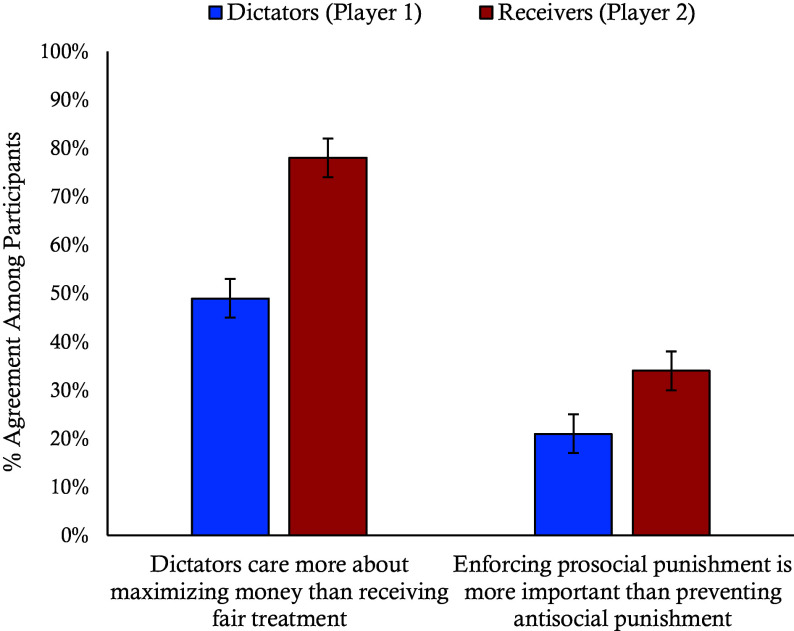
Perception of dictator preferences and punisher responsibilities by role. This graph shows the percentage of participants who believe dictators care more about maximizing money than receiving fair treatment, and whether enforcing prosocial punishment is more important than preventing antisocial punishment by role assignment from Experiment 8. Error bars represent SE.

Experiments 8 and 9 were preregistered replications of pilot experiments reported in the Supplemental Information. In Experiment 8, we examined how players’ roles may affect their perspectives when punishment is profitable by asking participants to imagine playing either as dictators or receivers in a paid punisher condition in a between-subjects experiment (N = 398). Participants who imagined themselves as dictators were significantly less trusting of punishers (M = 2.52, SD = 1.32) compared to those imagining themselves as receivers (M = 2.89, SD = 1.46), *F*(1, 396) = 7.24, *P* = 0.007, η^2^ = 0.02. Receivers (78%) were also more likely than dictators (49%) to believe that dictators prioritize maximizing the amount of money they make over being treated fairly χ^2^(1, 398) = 35.93, *P* < 0.001, φ = −0.30. Participants also differed in their views of punishers’ responsibilities. Receivers (34%) were more likely than dictators (21%) to state that it is more important in the game for punishers to punish unfair offers than to abstain from punishing fair offers, χ^2^(1, 397) = 8.11, *P* = 0.005, φ = −0.14 (Fig. 8).

**Fig. 9.**
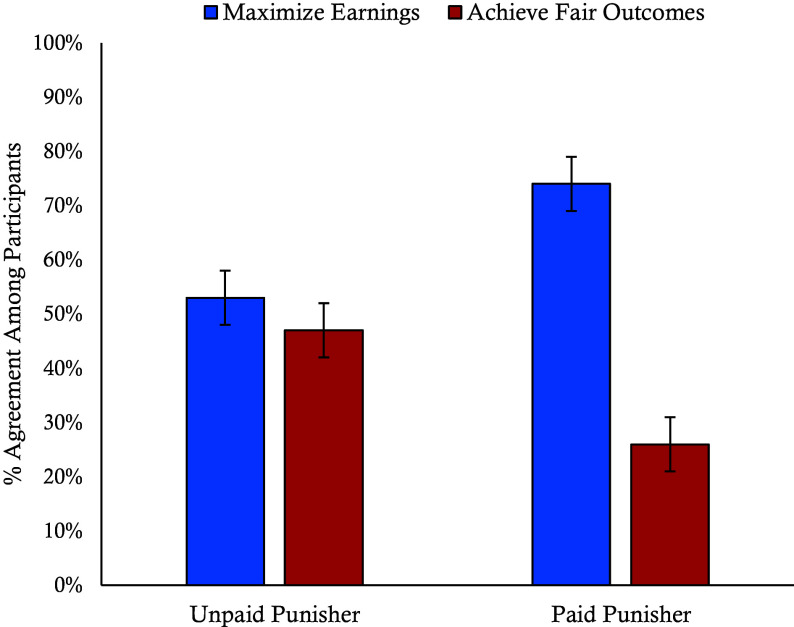
Perception of game purpose by incentive condition. This graph shows the percentage of participants who believe that the purpose of the game was to maximize earnings rather than achieve fair outcomes by incentive condition from Experiment 9. Error bars represent SE.

In Experiment 9, we investigated how perceptions of other players and social norms may change following the introduction of profit motives to punish in a between-subjects experiment wherein participants either imagined playing with a paid or unpaid punisher without specifying their role (N = 403). Participants who imagined playing in the condition where punishers are paid were only marginally less trusting of punishers (M = 3.20, SD = 1.29) compared to those who imagined playing in the unpaid condition (M = 3.42, SD = 1.35), *F*(1, 401) = 2.72, *P* = 0.10, η^2^ = 0.01, suggesting that naïve participants are unaware of the negative impact that payment has on dictators’ trust of punishers. When asked about the purpose of the game—whether it was about players maximizing their earnings or achieving a fair outcome—a significantly greater majority of participants in the paid condition (72%) believed the game was about maximizing earnings, compared to only 53% of participants in the unpaid condition, χ^2^(1, 403) = 14.32, *P* < 0.001, φ = 0.19, suggesting that the introduction of payment shifts perceptions of social norms toward self-interest (Fig. 9).

## Discussion

Some scholars have theorized that third-party punishment is a core mechanism through which large-scale cooperation emerges in social groups. However, across experiments, we find that when third-party punishment is profitable, rates of cooperation decrease. Reductions in cooperation occur prior to punishment exposure and persist following punishment, they do not fully recover even when punishment feedback is optimized for cooperative learning, and they are only prevented by restructuring the game so that only unfair offers can be punished. In spite of this, participants playing as receivers who materially benefit from increased cooperation disproportionately choose to play in conditions with paid punishers, reducing their own expected compensation by nearly 20% when compared to playing with an unpaid punisher or no punisher at all. Our judgment experiments suggest that receivers make suboptimal choices because most participants use a simple rational actor model in which payment should increase punishment and punishment should increase cooperation. Participants assigned to the receiver role are also more likely than dictators to trust paid punishers, to perceive dictators as motivated by monetary gain rather than a desire for fair treatment, and to value punishment of unfair offers over preventing punishment of fair offers. Finally, the introduction of monetary payment to engage in third-party punishment reframes perceptions of social norms such that participants are more likely to believe that the purpose of the game is for players to maximize their own earnings rather than to achieve a fair outcome for everyone.

Theoretically, our research highlights the key importance of communicative signaling in third-party punishment beyond the material costs it imposes on selfish actors (20–28). Punishment must communicate information about punisher’s intentions and social norms to effectively promote cooperation. When the communicative signals of punishment are degraded, such as through payment in our paradigm, people cannot reliably predict that fair behavior will go unpunished, social norms become ambiguous, and cooperation collapses. While we leveraged the use of profit motives to shift perceptions of punishment motives in our experiments, we believe our findings apply to other cases where motives may degrade the communicative signals of punishment, such as if people infer that punishers are motivated by racial bias or sadistic pleasure. Punishment may be an especially poor mechanism for promoting cooperation in social groups that are characterized by high levels of moral disagreement and competing values (30), as people will be especially likely to infer that punishers are driven by the wrong motives under these conditions. More generally, our findings suggest that psychological biases to overestimate the influence of self-interest on others’ behaviors (31) and to attribute malicious intentions to those who harm us (32) may create inherent challenges for punishment to effectively promote cooperation.

Explicitly eliminating the potential for antisocial behavior as we did in Experiment 4 may restore cooperation if signaled properly, echoing research that suggests that effective institutions can sustain cooperation through credible commitments in contexts where trust is compromised (33). However, these strategies create a second-order cooperation dilemma wherein social groups must enforce mechanisms to ensure that authorities and institutions act legitimately (34), or else individuals will be less motivated to cooperate themselves. For example, recent findings suggest that learning about insider trading by politicians reduces perceptions of congressional legitimacy and weakens citizens’ willingness to comply with the rule of law (35). More broadly, our findings contribute to ongoing debates about whether third-party punishment culturally evolved to support cooperation, and if not, what role it plays in the functioning of social groups. Recent research suggests that while common in laboratory settings, third-party punishment is rare in real-world contexts, especially prior to the emergence of modern states (36–39). Our findings suggest that even when third-party punishment is ineffective, social groups may nonetheless pursue it, raising the possibility that punishment culturally evolves in social groups due to a subjective rather than objective sense of its efficacy in promoting cooperation (40). Alternatively, punishment may have culturally evolved primarily to serve noncooperative purposes altogether, such as to facilitate competition, hierarchical control over subordinate groups, or the exclusion of undesired group members (41–42).

In terms of policy, our findings suggest that crime reduction strategies that draw on profit motives to increase punishment, such as by offering additional funding or bonuses to meet arrest or ticket quotas, may severely undermine cooperation. These strategies, which disproportionately target minority groups (43–44), may exacerbate distrust in law enforcement and reduce the willingness of criminalized communities to adhere to laws or cooperate with authorities (45–46). This is important to consider given the growing role of profit motives in administering punishment in the United States, with private prisons now housing over 8% of the incarcerated population (47), and civil asset forfeiture by law enforcement agencies exceeding the total value of all civilian theft and burglary (48). Once profit motives become culturally instantiated, their destructive effects on cooperation may persist even if authorities act appropriately, as people will be skeptical of authorities’ intentions and governing social norms unless structural reforms are explicitly signaled. Further, to the extent that noncriminalized communities trust and adopt the perspective of punitive authorities to a greater extent, they may rationally respond to any instances of crime by supporting profitable punishment and more severe penalties even as such policies backfire. In turn, political leaders may bolster their own support by advocating for more police and harsher penalties regardless of their effectiveness on crime reduction.

These results highlight the need to evaluate, reform, or possibly abolish institutional practices that compromise the communicative signaling function of punishment. Nonpunitive alternatives, such as restorative justice practices that seek to repair harm and relationships within the community, may prove more effective at fostering cooperation (49–50). While our findings provide evidence for the detrimental effects of profitable punishment on cooperation, limitations should be acknowledged. First, the third-party punishment game is a bargaining game, which may limit the generalizability of our findings. To explore whether our findings generalize to other kinds of situations, such as social dilemmas, we replicated our basic finding from Experiment 1 in a public goods game with punishment (See *SI Appendix* for Experiment 10). More broadly, our studies were conducted in controlled experimental settings that allow for precise manipulation of variables, but may not fully capture the complexity of real-world social interactions with institutions like law enforcement. In terms of causal mechanisms, we find evidence to suggest that profitable punishment destabilizes cooperation by signaling both an increased likelihood of antisocial punishment of fair behavior and that the social norms governing behavior in the game are selfish rather than cooperative. Punisher behavior and social norms are linked in real-world contexts, but future research should work to disentangle their relative contributions to decreased cooperation following the introduction of profit motives to punish. Further, while we focus on monetary profit motives, future research should explore how alternative nonmonetary profit motives, such as social disapproval or moral condemnation, interact with communicative signaling and cooperation (51). Finally, we conducted our studies exclusively with participants from the United States, which limits the cultural generalizability of our findings to other societies that may use different social heuristics for cooperation (52).

## Materials and Methods

All participants were recruited via Connect using CloudResearch and were prevented from participating in more than one study. CloudResearch offers enhanced participant vetting by blocking duplicate IP addresses, suspicious geolocation activity, and known bots. Participants in CloudResearch approved participants have been shown to demonstrate significantly better data quality, including more accurate responses and reduced rates of cheating compared to standard MTurk samples (53). Sample sizes were determined in advance using power analyses and are reported below with demographic information. Full materials, stimuli, and questionnaire items are provided in the *SI Appendix*. The research was approved by the UCSD Institutional Review Board, and informed consent was obtained from all participants.

**Experiment 1.** In a between-subjects design, 950 participants (Mage = 43; 489 female, 439 male, 7 nonbinary, 15 unreported) played a one-shot third-party punishment game as Player 1 (the dictator). Participants were randomly assigned to one of five conditions: no punisher, a paid punisher who could remove $5 or $10, or an unpaid punisher who could remove $5 or $10. They made a binary choice to either keep $20 or split it evenly with Player 2. Cooperation was measured as the sharing decision. Analyses used binary logistic regression.

**Experiment 2.** A total of 668 participants (Mage = 42; 282 male, 374 female, 8 nonbinary, 4 unreported) participated in a 12-round simultaneous third-party punishment game. It was a mixed design with within-subjects manipulation of punishment incentives and between-subjects manipulation of punishment order and severity. Players were randomly assigned to roles and experienced both paid and unpaid punishers in counterbalanced order across rounds 5 to 12 (rounds 1 to 4 featured no punishers). Punishers could remove either $5 or $10 from dictatorsand received a 5¢ bonus per punishment in paid rounds. Analyses used generalized linear mixed-effects models with random intercepts for participant.

**Experiment 3.** In a between-subjects design optimized for reinforcement learning, 994 participants (Mage = 42; 560 female, 421 male, 6 nonbinary, 7 unreported) played eight rounds of the profitable punishment game as dictators. Punishment was always prosocial (i.e., selfish offers were punished; fair offers were not), and participants were randomly assigned to either paid or unpaid punishers. The goal was to test whether learning from consistent punishment feedback would recover cooperation. Analyses used mixed-effects logistic regression.

**Experiment 4.** Participants (N = 1,011; 488 female, 510 male, 12 nonbinary, 1 unreported) played a one-shot game as dictators. In a 2 × 2 between-subjects design, punisher incentive was crossed (paid vs. unpaid) with punishment structure (punishment allowed only for selfish offers vs. always allowed). Cooperation was measured as the choice to split vs. keep the $20. Analyses used binary logistic regression.

**Experiment 5.** We conducted a preregistered internal meta-analysis on the dictator decisions from Studies 1 to 4 (N = 3,099; 12,395 total decisions). Cooperation was modeled as a function of punisher incentive using a generalized linear mixed-effects model with random intercepts for participant, round, and experiment.

**Experiment 6.** A total of 399 participants (Mage = 44; 193 female, 205 male, 1 nonbinary) played as receivers in a one-shot game and chose which punisher conditions they wished to play under (paid vs. unpaid, $10 severity vs. $5 severity, or no punisher). Participants were instructed to maximize their own monetary payoff by choosing the punisher type expected to increase cooperation. Expected earnings were calculated from Experiment 1 dictator behavior. Preferences were analyzed using binomial tests and chi-square comparisons.

**Experiment 7.** In a preregistered design, participants (N = 399; Mage = 44; 143 female, 134 male, 2 nonbinary, 7 unreported) received a description of the economic game and were asked about predicted behaviors of the players (e.g., likelihood of cooperation, punishment, and earnings) under various incentive conditions. Analyses used binomial tests comparing endorsement rates to chance.

**Experiment 8.** In a preregistered between-subjects design, 398 participants (Mage = 39; 187 female, 206 male, 3 nonbinary, 2 unreported) imagined playing the game as either the dictator or receiver. All participants were told that the punisher was paid. Measures included trust in the punisher, beliefs about dictator motives, and views of punisher responsibility. Analyses included an ANOVA and chi-square tests.

**Experiment 9.** In a preregistered between-subjects design, 403 participants (Mage = 43; 177 female, 211 male, 12 nonbinary, 6 unreported) read a vignette describing the game with either a paid or unpaid punisher (no role specified). Measures assessed trust in the punisher and perceptions of the game’s normative purpose. Analyses included an ANOVA and chi-square test.

## Supplementary Material

Appendix 01 (PDF)

## Data Availability

Anonymized All primary and processed data, code, results, and pre-registrations for studies 5, 6, 8 and 9 data have been deposited in OSF (https://osf.io/26tg3/files/osfstorage?view_only=aa00b86080c04696816e485c9ceaacf9) (54).
