# Supplementary material for: Profitable third-party punishment destabilizes cooperation
Source: Proc Natl Acad Sci U S A. 2025 Aug 19;122(34):e2508479122. doi: 10.1073/pnas.2508479122 (PMC12403122; doi:10.1073/pnas.2508479122)
Supplement: Supplementary file 1 — Appendix 01 (PDF) [file pnas.2508479122.sapp.pdf]

Supplementary Information

Experiment 1: Player 1 One-shot Game

We recruited participants from Amazon's Mechanical Turk (n = 950; M_{age} = 43; 489 females, 439 males, 7 non-binary, 15 missing) and paid them $.25. Participants were told that they would be playing a game involving three participants and that their decisions in the game were binding such that they would be played out for real money if selected by lottery. All participants played the game as Player 1, the dictators, and we manipulated the type of Player 3, the punisher, they were told they would play with. They were randomly assigned one of five conditions: No Player 3 (n = 190), Incentivized Player 3 who can remove $5 ($n$ = 196), Incentivized Player 3 who can remove $10 ($n$ = 188), Non-incentivized Player 3 who can remove $5 ($n$ = 190), or Non-incentivized Player 3 who can remove $10 ($n$ = 186). All participants were then asked to make the binary choice between "Keep $20 to yourself and allocate $0 to Player 2" or "Keep $10 to yourself and allocate $10 to Player 2." This was the binary choice in all experiments involving Player 1 decisions.

For the analyses, we first combined the unpaid punisher conditions (Unpaid Player 3 who can remove $5 and Unpaid Player 3 who can remove $10) and the paid punisher conditions (Paid Player 3 who can remove $5 and Paid Player 3 who can remove $10) and then compared each of those conditions to the no punisher control condition using a binary logistic regression. We found that the introduction of monetary incentives to punish significantly reduced dictators' willingness to cooperate (β = -.39, SE = .18, z = -2.19, p = .029), while the introduction of non-incentivized punishment did not significantly change dictators' behavior (β = .04, SE = .18, z = -.20, p = .839), compared to when no punisher was present. We then conducted a second binary logistic regression model predicting cooperation from payment condition and punishment severity ($5 or $10), including only the four punisher conditions. We did this to test whether the effects would appear controlling for punishment severity. Analyses revealed a main effect of financial incentives (β = -.43, SE = .15, z = -2.93, p = .003) such that participants were less likely to cooperate when punishers received a bonus payment for punishing. There was no main effect of punishment severity (β = -.04, SE = .15, z = -.27, p = .785).

Game Instructions for Paid Player 3 Conditions

How the game works:

In the game, Player 1 is provided with $20 dollars. ***Player 1 can either keep the entire $20 for themselves, or they can keep $10 and allocate the other $10 dollars to Player 2.***

Following Player 1's decision, ***Player 3 can earn a bonus payment by eliminating [$5/$10] from Player 1's final amount regardless of their decision.*** *While Player 3 keeps the bonus payment, Player 3 does not get to keep the eliminated [$5/$10] for themselves, nor does the [$5/$10] go to Player 2; the [$5/$10] is simply eliminated from whatever Player 1's final amount is.*

You are Player 1. *You must decide whether to keep the entire $20 for yourself or whether to allocate $10 to Player 2.*

Player 3 will learn of your choice and then make their decision about whether to earn a bonus payment by removing [$5/$10] from you.

All decisions are binding. *Once all participants have made their decisions, they may be randomly selected to have their game played out for actual money as part of a lottery.*

You are Player 1. *You have been allocated $20. If selected via lottery, do you wish to keep $20 to yourself and allocate $0 to Player 2 or do you wish to keep $10 to yourself and allocate $10 to Player 2? Remember that Player 3 can choose to earn a bonus payment and eliminate [$5/$10] from your final amount if they wish to.*

<u>Game Instructions for Unpaid Player 3 Conditions</u>

How the game works:

In the game, Player 1 is provided with $20 dollars. ***Player 1 can either keep the entire $20 for themselves, or they can keep $10 and allocate the other $10 dollars to Player 2.***

Following Player 1's decision, ***Player 3 can choose to eliminate [$5/$10] of the money that Player 1 would end up with regardless of what Player 1 decides.*** *Player 3 does not get to keep the eliminated [$5/$10] for themselves, nor does the [$5/$10] go to Player 2; the [$5/$10] is simply eliminated from whatever Player 1's final amount is.*

You are Player 1. *You must decide whether to keep the entire $20 for yourself or whether to allocate $10 to Player 2.*

Player 3 will learn of your choice and then make their decision *about whether to remove [$5/$10] from you.*

All decisions are binding. *Once all participants have made their decisions, they may be randomly selected to have their game played out for actual money as part of a lottery.*

You are Player 1. *You have been allocated $20. If selected via lottery, do you wish to keep $20 to yourself and allocate $0 to Player 2 or do you wish to keep $10 to yourself and allocate $10 to Player 2? Remember that Player 3 can choose to eliminate [$5/$10] from your final amount if they wish to.*

<u>Game Instructions for No Punisher Condition</u>

How the game works:

In the game, Player 1 is provided with $20 dollars. ***Player 1 can either keep the entire $20 for themselves, or they can keep $10 and allocate the other $10 dollars to Player 2.***

You are Player 1. *You must decide whether to keep the entire $20 for yourself or whether to allocate $10 to Player 2.*

All decisions are binding. *Once all participants have made their decisions, they may be randomly selected to have their game played out for actual money as part of a lottery.*

You are Player 1. *You have been allocated $20. If selected via lottery, do you wish to keep $20 to yourself and allocate $0 to Player 2 or do you wish to keep $10 to yourself and allocate $10 to Player 2?*

<u>Experiment 1: Player 3 One-shot Game</u>

We recruited participants from Amazon’s Mechanical Turk (*n* = 1248; M_{age} = 41; 588 females, 616 males, 12 non-binary, 32 missing) and paid them $.25. Participants were told that they would be playing a game involving three participants and that their decisions in the game were binding such that they would be played out for real money if selected by lottery. All participants played the game as Player 3, the punishers, who could remove $5 from Player 1. We manipulated the amount of payment they would receive for engaging in punishment. They were randomly assigned one of three conditions: unpaid (*n* = 417), one-cent payment (*n* = 413), or 5-cent payment (*n* = 418). Participants in all conditions made two binding choices about whether they would eliminate money from Player 1 if Player 1 kept all of the money (prosocial punishment) and if Player 1 split the money (antisocial punishment).). Specifically, participants were then to make the binary choice between “Yes, eliminate $5 from Player 1” or “No, do not eliminate $5 from Player 1.”

We conducted chi square tests to examine punishment rates between the conditions. The results revealed a significant association between incentives and prosocial punishment, χ^2 (2, 1240) = 12.373, *p* = .002, φ = .10. Replicating prior work (Rai, 2022), post-hoc pairwise comparisons showed that participants were significantly less likely to engage prosocial punishment in the 1-cent payment condition (41%) compared to the non-incentivized punishment condition (51%) (χ^2 (1, 824) = 7.512, *p* = .006, φ = -.095) and the 5-cent payment condition (52%) (χ^2 (1, 826) = 10.874, *p* < .001, φ = -.115). There was no significant difference in prosocial punishment rates between the non-incentivized and 5-cent payment conditions χ^2 (1, 830) = .307, *p* = .58. The results also revealed a significant association between incentives and antisocial punishment, χ^2 (2, 1248) = 16.553, *p* < .001, φ = .115. Post-hoc pairwise comparisons showed that participants were significantly more likely to engage in antisocial punishment in the 5-cent payment condition (26%) compared to the one-cent payment condition (19%) χ^2 (1, 831) = 4.963, *p* = .026, φ = -.077, and the non-incentivized condition (15%) χ^2 (1, 835) = 16.247, *p* < .001, φ = .139. The difference in antisocial punishment rates between the non-incentivized and 1-cent payment condition was marginal χ^2 (1, 830) = 3.309, *p* = .069.

<u>Game Instructions for Paid Player 3 Conditions</u>

How the game works:
In the game, Player 1 is provided with $20 dollars. ***Player 1 can either keep the entire $20 for themselves, or they can split the money by keeping $10 for themselves and allocating the other $10 dollars to Player 2.***

Following Player 1's decision, ***Player 3 can earn a [small/moderate] bonus of [1/5] [cent/cents] by eliminating $5 of the money that Player 1 wound up with regardless of what Player 1 decides.*** *While Player 3 keeps the [small/moderate] bonus of* ***[1/5] [cent/cents]****, Player 3 does not get to keep the eliminated $5 for themselves, nor does the $5 go to Player 2; the $5 is simply eliminated from whatever Player 1's final amount is.*

You are Player 3. *You must decide whether to eliminate $5 from Player 1.*

All decisions are binding. *Once all participants have made their decisions, they may be randomly selected to have their game played out for actual money as part of a lottery. By random assignment, approximately 1 in 20 sets of participants will be selected.*

<u>Prosocial Punishment Decision</u>

You are Player 3. *If selected via lottery, do you wish to* ***earn a [small/moderate] bonus of [1/5] [cent/cents]*** *by eliminating $5 from Player 1* ***if Player 1 chooses to keep the entire $20 to themselves and allocate nothing to Player 2?***

Remember, the $5 does not go to Player 2 or yourself, it is simply eliminated from Player 1.

<u>Antisocial Punishment Decision</u>

You are Player 3. *If selected via lottery, do you wish to* ***[small/moderate] a small bonus of [1/5] [cent/cents]*** *by eliminating $5 from Player 1* ***if Player 1 chooses to split the money by keeping $10 for themselves and allocating $10 to Player 2?***

Remember, the $5 does not go to Player 2 or yourself, it is simply eliminated from Player 1.

<u>Game Instructions for Unpaid Player 3 Conditions</u>

How the game works:

In the game, Player 1 is provided with $20 dollars. ***Player 1 can either keep the entire $20 for themselves, or they can split the money by keeping $10 for themselves and allocating the other $10 dollars to Player 2.***

Following Player 1's decision, ***Player 3 can choose to eliminate $5 of the money that Player 1 would end up with regardless of what Player 1 decides.*** *Player 3 does not get to keep the eliminated $5 for themselves, nor does the $5 go to Player 2; the $5 is simply eliminated from whatever Player 1's final amount is.*

You are Player 3. *You must decide whether to eliminate $5 from Player 1.*

All decisions are binding. *Once all participants have made their decisions, they may be randomly selected to have their game played out for actual money as part of a lottery. By random assignment, approximately 1 in 20 sets of participants will be selected.*

Prosocial Punishment Decision

You are Player 3. *If selected via lottery, do you wish to eliminate $5 from Player 1* ***if Player 1 chooses to keep the entire $20 to themselves and allocate nothing to Player 2?***

Remember, the $5 does not go to Player 2 or yourself, it is simply eliminated from Player 1.

Antisocial Punishment Decision

You are Player 3. *If selected via lottery, do you wish to eliminate $5 from Player 1* ***if Player 1 chooses to split the money by keeping $10 for themselves and allocating $10 to Player 2?***

Remember, the $5 does not go to Player 2 or yourself, it is simply eliminated from Player 1.

Experiment 2: Simultaneous Repeated Game

We recruited participants from Connect and paid them $1.00 to play the economic game for 12-rounds using SMARTRIQS (Molnar, 2019), an open-source platform that allows for interactive experiments via Qualtrics. Since interactive experiments can face high levels of attrition (Arechar et al., 2018), where inactivity from one participant can disrupt game sessions, we recruited large samples of participants. To minimize the impact of attrition, we grouped participants into sets of four, with each group consisting of two Player 1s and two Player 3s. Within each group, participants were told they would interact with a new individual every round. In reality, they alternated between the same two partners across rounds. For example, in a group with participants A, B, C, and D, A and B played as Player 1s, while C and D played as Player 3s. In one round, A would play with C and B with D, and in the next round, A would play with D and B with C. This setup ensured that only four active participants were needed to complete the game and collect viable data. In total, we recruited 1,662 participants. However, 994 data points were excluded due to incomplete games, where one or more participants in a group failed to complete all rounds, or due to participants not being paired with other players at all. After these exclusions, we retained 668 participants, 334 Player 1 dictators and 334 Player 3 punishers (M_{age} = 42, 282 males, 374 females, 8 non-binary, 4 missing).

Participants were told that they would be playing a game for multiple rounds involving three participants and that their decisions were binding such that they would be played out for real money if selected by lottery. The game was divided into three phases: in the first four rounds, Player 1s made allocation decisions without the presence of Player 3s, who answered filler questions during this phase. In the next eight rounds, we introduced Player 1s and Player 3s to each other. Here, participants experienced both paid and unpaid punisher conditions in randomized order: some groups experienced paid punishers in rounds 5–8 followed by unpaid punishers in rounds 9–12, while others experienced the reverse. Punishment severity was also manipulated, such that punishers could remove either $5 or $10 from Player 1 dictators following

their allocation decisions. Among the 334 Player 3s, 188 were assigned to the $10 punishment condition, and 146 were assigned to the $5 punishment condition, with the same distribution applying to their paired Player 1s (see Supplementary Table 1 for a breakdown of condition assignments). Player 1s were informed about whether they were playing with paid or unpaid Player 3s but were not told the exact bonus payment paid Player 3s would receive, while paid Player 3s were told they would be earning a 5-cent bonus if they engaged in punishment.

For the analyses, we first examined cooperation rates over time without punishers by aggregating cooperation rates across all sequences and punishment severity conditions from rounds 1 to 4. A generalized binomial linear mixed-effects model with round number as a fixed effect and participant included as a random effect showed that cooperation significantly declined across rounds ($\beta = -.82$, $p < .001$). To examine how the introduction of paid and unpaid punishment affects cooperation, we pooled cooperation counts from specific rounds to represent paid and unpaid punisher conditions. For the paid punisher condition, we included rounds 5-8 for the punisher condition sequence that went from paid to unpaid punishment and rounds 9-12 when the sequence went from unpaid to paid punishment. For the unpaid punisher condition, we included rounds 5-8 when the punisher condition sequence was unpaid to paid punishment and rounds 9-12 when the sequence was paid unpaid punishment. The baseline condition was defined as the cooperation rate in the last round without punishers (round 4).

First, to examine cooperation after introducing paid and unpaid punishers, we used a generalized binomial linear mixed-effects model predicting cooperation from condition (baseline, paid, unpaid) with random effects for participants. Results revealed a significantly lower likelihood of cooperation in the paid condition compared to the baseline ($\beta = -1.81$, $SE = .20$, $z = -9.02$, $p < .001$), but no significant difference between unpaid punishers and baseline ($\beta = -.12$, $SE = .19$, $z = -.61$, $p = .54$). The probability of cooperating in the baseline condition (last round without a punisher) was 72% (SE = 6.1%), while it was 69% (SE = 5.5%) in the unpaid punisher condition, and only 29% (SE = 5.4%) in the paid punisher condition. We ran the same model including an effect of punisher sequence (paid to unpaid or unpaid to paid) to control for order effects. Results again revealed a significantly lower likelihood of cooperation in the paid condition compared to the baseline ($\beta = -1.81$, $SE = .20$, $z = -9.02$, $p < .001$), but no significant difference between unpaid punishers and baseline ($\beta = -.12$, $SE = .19$, $z = -.61$, $p = .54$). The effect of punisher sequence was only marginal ($\beta = -.91$, $SE = .50$, $z = -1.82$, $p = .07$), suggesting that playing with a paid punisher first may reduce overall cooperation. We also tested interactions with punishment severity. We ran a generalized binomial linear mixed-effects model predicting cooperation by condition, punishment severity ($5 or $10) and their interaction, with random effects for participants. Results revealed no main effect of punishment severity ($\beta = .51$, $SE = .60$, $z = .85$, $p = .40$). We also found no significant interaction between severity and the paid condition ($\beta = -.69$, $SE = .40$, $z = -1.70$, $p = .09$) nor for severity and the unpaid condition ($\beta = .23$, $SE = .39$, $z = -.59$, $p = .55$). We also examined how cooperation changed over time in the paid and unpaid conditions separately using a generalized binomial linear mixed-effects model with round number as a fixed effect and random effects for participants. We found that cooperation remained stable over time in the paid condition ($\beta = -.004$, $SE = .085$, $z = -.043$, $p = 0.97$), while it declined overtime in the unpaid condition ($\beta = .215$, $SE = .100$, $z = -2.145$, $p = .032$). See Supplementary Figure 1 for raw cooperation rates from rounds 1-12 between punisher sequence orders.

Next, we analyzed prosocial and antisocial punishment rates. Antisocial punishment was coded as instances where Player 1s transferred $10 but received a punishment from Player 3. Prosocial punishment was coded as instances where participants transferred $0 and received a punishment from Player 3. Counts of antisocial and prosocial punishment were then aggregated by summing the occurrences of each punishment type across rounds. These counts were divided by the total number of opportunities for each punishment type to calculate proportions. For antisocial punishment, opportunities were defined as the number of cooperative transfers, while for prosocial punishment, opportunities were defined as the number of non-cooperative transfers.

We compared proportions of antisocial and prosocial punishments between paid and unpaid conditions using generalized binomial linear mixed-effects models with random effects for participants. We found that paid punishers were significantly more likely to engage in prosocial punishment (77%, SE = 2.27%) than unpaid punishers (52%, SE = 3.27%; $\beta = 1.14$, $p < .001$), and significantly more likely to engage in antisocial punishment (14%, SE = 2.71%) than unpaid

punishers (2.4%, SE = .71%; β = 1.84, p < .001). We ran the same model including an effect of payment sequence (paid to unpaid or unpaid to paid) to control for order effects. For antisocial punishment, the results revealed a significant effect of incentive condition, such that the likelihood of antisocial punishment was significantly higher in the paid condition (β = 1.84, SE = .23, z = 8.13, p < .001), but there was no effect of payment sequence (β = .17, SE = .36, z = .47, p = .64). For prosocial punishment, the model also revealed a significant effect of condition, with prosocial punishment being significantly higher in the paid condition (β = 1.14, SE = .14, z = 8.19, p < .001), but there was no effect of payment sequence (β = .05, SE = .21, z = .24, p = .81). We also tested interactions with punishment severity. We ran a generalized binomial linear mixed-effects model predicting antisocial and prosocial punishment likelihoods (separately) by incentive condition, punishment severity ($5 or $10) and their interaction, with random effects for participants. For antisocial punishment, results revealed no main effect of punishment severity (β = -.38, SE = .39, z = .99, p = .32), but there was a significant interaction between severity and incentive condition (β = .85, SE = .43, z = 1.98, p = .048). Follow up analyses revealed that increasing punishment severity reduced the likelihood of antisocial punishment in the paid condition but increased it in the unpaid condition. For prosocial punishment, results revealed a main effect of punishment severity (β = -.56, SE = .25, z = -2.21, p = .03), such that the likelihood of prosocial punishment was lower in the $10 compared to $5 severity condition. However, there was no significant interaction between severity and incentive conditions (β = .23, SE = .28 z = .84, p = .40). Next, we ran a generalized binomial linear mixed-effects model predicting punishment by condition, punishment type (prosocial vs. antisocial), and their interaction, with random effects for participants. Results from the generalized binomial linear mixed-effects model revealed a significant main effect of condition (β = -1.10, SE = .14, z = -8.05, p < .001) and punishment type (β = -2.58, SE = .18, z = -14.17, p < .001), such that there was greater punishment in the paid condition than unpaid condition, and there was greater prosocial than antisocial punishment. We also found a marginal interaction between condition and punishment type (β = -.43, SE = .23, z = -1.84, p = .065), suggesting that the increase in punishment under the paid condition was more pronounced for prosocial than antisocial punishment. See Supplementary Figures 2 and 3 for raw punishment rates from rounds 5-12 between punisher sequence orders.

Game Instructions for All Conditions

How the game works:

In the game, Player 1 is provided with $20 dollars. ***Player 1 can either keep the entire $20 for themselves, or they can keep $10 and allocate the other $10 dollars to Player 2.***

Following Player 1's decision, ***Player 3 can eliminate [$5/$10] from Player 1's final amount regardless of their decision.*** *Player 3 does not get to keep the eliminated [$5/$10] for themselves, nor does the [$5/$10] go to Player 2; the [$5/$10] is simply eliminated from whatever Player 1's final amount is.*

In some rounds, Player 3 earns a bonus payment if they eliminate [$5/$10] from Player 1. *Players will be notified when these changes happen in a round before they make their choices.*

This game will be repeated. In each round, you will be grouped with new participants, ensuring that each interaction is with different people from the previous rounds.

All decisions are binding. *Once all participants have made their decisions, some participants may be randomly selected to have their games played out for actual money as part of a lottery.*

Game Instructions for Player 1 Rounds 1-4

You are Player 1. *To start, for these first 4 rounds, you will be playing this game only with Player 2.* ***There is no Player 3 in the first four rounds****.*

You are Player 1. *If selected via lottery, do you wish to keep $20 to yourself and allocate $0 to Player 2 or do you wish to keep $10 to yourself and allocate $10 to Player 2?*

There is no Player 3 in this round.

Filler Instructions for Player 3 Rounds 1-4

Player 3 was told, "Before the game begins, we want you to answer a few questions. Please move forward with the survey." They were then asked to answer yes or no to whether they read every day, exercise every day, drive every day, and listen to music every day.

Incentive Introduction Instruction for Player 1 and Player 3

Now, in these next four rounds, there is a ***Player 3. Player 3 earns a bonus payment if they eliminate [$5/$10] from Player 1.***

Player 1 Instructions in rounds with Paid Player 3

You are Player 1. *If selected via lottery, do you wish to keep $20 to yourself and allocate $0 to Player 2 or do you wish to keep $10 to yourself and allocate $10 to Player 2?*

Remember that Player 3 can earn a bonus payment and choose to eliminate [$5/$10] from your final amount if they wish to.

Player 3 Instructions in Profitable Rounds

Player 1 has transferred [0/10] dollars to Player 2. Player 1 kept [10/20] dollars.

If selected via lottery, do you wish to ***earn a moderate bonus of 5 cents*** *by eliminating [$5/$10] from Player 1?*

Remember, the ***[$5/$10]*** *does not go to Player 2 or yourself, it is simply eliminated from Player 1.*

Unpaid Introduction Instruction for Player 1 and Player 3

Now, for the last four rounds, ***Player 3 does NOT earn a bonus payment if they eliminate [$5/$10] from Player 1.***

Player 1 Instructions in rounds with Unpaid Player 3

You are Player 1. *If selected via lottery, do you wish to keep $20 to yourself and allocate $0 to Player 2 or do you wish to keep $10 to yourself and allocate $10 to Player 2?*

Remember that Player 3 can choose to eliminate [$5/$10] from your final amount if they wish to.

Player 3 Instructions in Unpaid Rounds

Player 1 has transferred [0/10] dollars to Player 2. Player 1 kept [10/20] dollars.

If selected via lottery, do you wish to eliminate [$5/$10] from Player 1?

Remember, the [$5/$10] does not go to Player 2 or yourself, it is simply eliminated from Player 1.

Player 1's Instructions after Decision

You transferred [$10/$0] to Player 2 and kept [$10/$20] for yourself.

Now Player 3 will make a decision whether to reduce your payment or not. On the next screen, you will have to wait until Player 3 makes a decision.

Player 1's Instructions after Player 3's Decision

You transferred $[10/0] dollars to Player 2.
You kept $20 – [$10/$0} =[$10/$20] dollars.

Player 3 decided to reduce your payment by $[10/5] dollars.

Your final payoff is $[20/15/10/5/0] dollars.

Experiment 3: Repeated Game Only Prosocial Punishment

We recruited participants from Amazon's Mechanical Turk and Connect (n = 994; M_{age} = 42; 560 females, 421 males, 6 non-binary, 7 missing) and compensated Mechanical Turk workers $0.25 and Connect workers $0.50. We recruited participants from Connect as well because our Mechanical Turk pool was shrinking due to the number of game experiments we had conducted on the platform. Participants were told that they would be playing a repeated game involving three participants and that their decisions were binding such that they would be played out for real money if selected by lottery. All participants played the game as Player 1, the dictators, and we manipulated the type of Player 3, the punisher, they were told they would play with. They were randomly assigned to play with a paid (n = 497) or unpaid (n = 497) Player 3 who can remove $10 and were given the same instructions as in Experiments 1 and 2. In reality, the experiment was programmed so that participants were always punished if they kept all of the money (prosocial punishment) and never punished for sharing the money with recipients (antisocial punishment).

For the analyses, we examined the effect of round number, condition (paid vs. unpaid) and their interaction on cooperation using a generalized binomial linear mixed-effects model with random effects for participants. The analysis revealed a significant main effect of round (β = .15, SE = .02, z = 6.19, p < .001), indicating that cooperation increased across rounds. There was also a significant main effect of condition, with higher cooperation rates in the unpaid punisher condition compared to the paid punisher condition (β = 1.28, SE = .34, z = 3.77, p < .001). However, the interaction between round and condition was not significant (β = -.02, SE = .03, z = -.63, p = .53).

Instructions if Player 1 Did Cooperate

You transferred 10 dollars to Player 2 and kept 10 dollars.

Player 3 decided not to reduce your payment.

Your final payoff is 10 dollars.

Instructions if Player 1 Did Not Cooperate

You transferred 0 dollars to Player 2 and kept 20 dollars.

Player 3 decided to reduce your payment by 10 dollars.

Your final payoff is 10 dollars.

Experiment 4: One-shot Game with Altered Punishment Structure

We recruited participants from Connect (n = 1,011; 488 females, 510 males, 12 non-binary, 1 missing; age data was not collected due to an oversight) and compensated them $0.50 for their participation. The experiment used a 2 (incentive: yes vs. no) × 2 (altered punishment structure: yes vs. no) between-subjects design. Here, Player 3 could eliminate $10 and the instructions for the incentive conditions were identical to those in Experiments 1-3. In the altered punishment structure conditions, participants were informed that Player 3 could only eliminate money if Player 1 did not share money with Player 2. This created four experimental conditions: unpaid with altered punishment structure (n = 254), unpaid without altered punishment structure (n = 252), paid with altered punishment structure (n = 251), and paid without altered punishment structure (n = 254).

For the analyses, we conducted a binary logistic regression to examine the effects of altered punishment structure, incentives, and their interactions on cooperation. The analysis revealed a main effect of incentive ($\beta = -.72$, $SE = .18$, $z = -3.93$, $p < .001$) such that Player 1s cooperated more with paid vs. unpaid Player 3s. There was also a main effect of altered punishment structure ($\beta = 1.18$, $SE = .19$, $z = 6.17$, $p < .001$) such that Player 1s cooperated more when Player 3 could only punish unfair offers. Most importantly, we found a significant interaction such that eliminating the potential for antisocial punishment (i.e., altered punishment structure condition) had a stronger effect on increasing cooperation when punishers were paid versus not paid ($\beta = -0.76$, $SE = .27$, $z = -2.79$, $p = .005$).

<u>Game Instructions for Paid Punisher with Altered Punishment Structure</u>

How the game works:

In the game, Player 1 is provided with $20 dollars. ***Player 1 can either keep the entire $20 for themselves, or they can keep $10 and allocate the other $10 dollars to Player 2.***

If Player 1 keeps all the money rather than sharing it with Player 2, ***Player 3 can earn a bonus payment by eliminating $10 from Player 1's final amount.*** *While Player 3 keeps the bonus payment, Player 3 does not get to keep the eliminated $10 for themselves, nor does the $10 go to Player 2; the $10 is simply eliminated from whatever Player 1's final amount is.*

You are Player 1. *You must decide whether to keep the entire $20 for yourself or whether to allocate $10 to Player 2.*

Player 3 will learn of your choice and then if you decide to keep the entire $20, they can choose to earn a bonus payment by removing $10 from you.

All decisions are binding. *Once all participants have made their decisions, they may be randomly selected to have their game played out for actual money as part of a lottery.*

You are Player 1. *You have been allocated $20. If selected via lottery, do you wish to keep $20 to yourself and allocate $0 to Player 2 or do you wish to keep $10 to yourself and allocate $10 to Player 2? Remember that if you keep the entire $20 to yourself, Player 3 can choose to earn a bonus payment and eliminate $10 from your final amount if they wish to.*

<u>Game Instructions for Unpaid Punisher with Altered Punishment Structure</u>

How the game works:

In the game, Player 1 is provided with $20 dollars. ***Player 1 can either keep the entire $20 for themselves, or they can keep $10 and allocate the other $10 dollars to Player 2.***

If Player 1 keeps all the money rather than sharing it with Player 2, ***Player 3 can choose to eliminate $10 of the money that Player 1 would end up with.*** *Player 3 does not get to keep the eliminated $10 for themselves, nor does the $10 go to Player 2; the $10 is simply eliminated from whatever Player 1's final amount is.*

You are Player 1. *You must decide whether to keep the entire $20 for yourself or whether to allocate $10 to Player 2.*

Player 3 will learn of your choice and then if you decide to keep the entire $20, they can choose to remove $10 from you.

All decisions are binding. *Once all participants have made their decisions, they may be randomly selected to have their game played out for actual money as part of a lottery.*

You are Player 1. *You have been allocated $20. If selected via lottery, do you wish to keep $20 to yourself and allocate $0 to Player 2 or do you wish to keep $10 to yourself and allocate $10 to Player 2? Remember that if you keep the entire $20 to yourself, Player 3 can eliminate $10 from your final amount if they wish to.*

Experiment 5: Internal Meta-Analysis

We conducted an internal meta-analysis using data from Experiments 1 through 4 to examine whether the effect of paid punishment on cooperation was robust across the different experimental designs. All included studies featured dictator decisions about whether to cooperate by splitting the $20 endowment, with a third-party punisher present who was either paid or not. The data included a total of 3,099 unique participants and 12,395 cooperation decisions, drawn from one-shot games (Experiment 1 and 4) and repeated rounds (Experiments 2 and 3). We fit a generalized linear mixed-effects model using the glmer() function in the lme4 package in R. The dependent variable was binary cooperation (1 = split money, 0 = keep all), and the model included punishment condition (condition_incentive, coded as Paid or Unpaid) as a fixed effect. We included random intercepts for participant_id, round, and experiment_id to account for repeated measures and experiment-level differences. As pre-registered, the final model specification was:

cooperation ~ condition_incentive + (1 | participant_id) + (1 | round) + (1 | experiment_id)

The model revealed a significant effect of condition, such that participants were less likely to cooperate when punishers were paid (β = -1.62, SE = .13, z = -12.92, p < .001). Model-estimated probabilities indicated that the likelihood of cooperation was 58% (SE = .09, 95% *CI*: [41%, 74%]) in the paid condition compared to 88% (SE = .04, 95% *CI*: [78%, 93%]) in the unpaid condition.

Experiment 6: Player 2 One-shot Game Decisions

We recruited participants from Amazon's Mechanical Turk (n = 399; M_{age} = 44; 193 females, 205 males, 1 non-binary) and paid them $.25. Participants were told that they would be playing a game involving three participants and that their decisions were binding such that they would be played out for real money if selected by lottery. All participants played the game as Player 2, the receiver. They were told that they could choose conditions to play the game with the goal of trying to maximize how much money Player 1 would give them. They were asked whether they wanted to play with a Player 3 at all (yes or no), whether they wanted to incentivize Player 3 (yes or no), and whether they would want Player 3 to be able to remove $5 or $10 ($10 or $5).

We conducted binomial tests to assess whether participants' responses significantly deviated from chance (.50). Results revealed that most participants chose to play with a punisher (67%, SE = 2%; p < .001). Then we examined preferences for incentives and severity among the participants that chose to play with a Player 3 punisher (n = 268). We found that most who decided to play with a punisher chose incentivize punishers (64%, SE = 3%; p < .001) and most chose more severe punishment (59%, SE = 3%; p = .003). Lastly, we examined preferences for severity among the participants that chose to incentivize Player 3 (n = 171). We found that most participants who decided to incentivize punishers chose more severe punishment (61% SE = 4%; p = .004).

Game Instructions for Player 2

How the game works:

In the game, Player 1 is provided with $20 dollars. ***Player 1 can either keep the entire $20 for themselves, or they can keep $10 and allocate the other $10 dollars to Player 2.***

After observing Player 1's decision, ***Player 3 can choose to eliminate some of the money that Player 1 would end up with regardless of what Player 1 decides.*** *Player 3 does not get to keep the eliminated money for themselves, nor does the money go to Player 2; the money is simply eliminated from whatever Player 1's final amount is.*

You are Player 2. *You cannot affect or change Player 1's decision about whether to share the money with you on your own. However, as Player 2, you are allowed to make decisions about game conditions that affect Players 1 and 3.*

Your goal is to make choices that will influence Player 1 to share with you to maximize the money you receive in the game. *The more accurate you are in choosing, the more money you will make.*

All decisions are binding. *Once all participants have made their decisions, some participants may be randomly selected to have their games played out for actual money as part of a lottery.*

Questions for Player 2

You can choose whether the experimenters should include a Player 3 who can eliminate money from Player 1 after observing their decision toward you.

Would you like there to be a Player 3 who can decide to eliminate money from Player 1 after observing their decision toward you?

You can choose whether the experimenters should incentivize Player 3 to eliminate money from Player 1 after observing their decision toward you.

Would you like Player 3 to receive a bonus payment from the experimenters if they eliminate money from Player 1 after observing their decision toward you?

You can choose how much money Player 3 can eliminate from Player 1 after observing their decision toward you.

Would you like Player 3 to be able to eliminate $10 or $5 from Player 1 after observing their decision toward you?

Experiment 7: Naïve Participant Perceptions of the Game

We recruited participants from Amazon's Mechanical Turk (n = 399; M_{age} = 44; 143 females, 134 males, 2 non-binary, 7 missing) and paid them $.25. Participants were told that they would learn about a game and then answer questions about it. They were asked whether they thought Player 1 would be more likely to share with Player 2 in the presence of a Player 3 who can remove money (more likely or less likely), whether Player 3 is more likely to punish if they received a bonus for doing so (more likely or less likely), whether Player 2 would earn more money if Player 3 received a bonus for punishing (more money or less money), and whether Player 1 would be more likely to share money if Payer 3 could remove $10 or 5$ ($10 or 5$). We conducted binomial tests to assess whether participants' responses significantly deviated from chance (.50). Results showed that 85% (SE = 2%) of participants believed that dictators would be more likely to share money with recipients if a punisher was present (p < .001) and 89% (SE = 2%) of participants believed that the punisher would be more likely to eliminate money from dictators if they received a bonus for doing so (p < .001). Consequently, most participants (63%; SE = 3%) believed that recipients would receive more money if they played with a paid punisher (p < .001). Additionally, we found that 66% (SE = 3%) of participants believed that more severe punishment would increase cooperation (p < .001).

Questions

In the game, sometimes there is a Player 3 who can remove money from Player 1 after observing their decision toward Player 2.

Do you think Player 1 is more or less likely to share the money with Player 2 if Player 3 can remove money from them after observing their decision toward Player 2?

In the game, sometimes the experimenters give Player 3 a bonus for removing money from Player 1 after observing their decision toward Player 2.
Do you think Player 3 is more or less likely to remove money from Player 1 after observing their decision toward Player 2 if they receive a bonus for doing so?
Do you think Player 2 will make more or less money if Player 3 receives a bonus from the experimenters for removing money from Player 1 after observing their decision toward Player 2?

In the game, sometimes Player 3 can remove $5 from Player 1, and other times they can remove $10 from Player 1.
Do you think Player 1 will be more likely to share the money with Player 2 if Player 3 can remove $5 from them or $10 from them?

Experiment 8 Pilot: Player 1 and Player 2 Perceptions of Paid Punishment Condition

We recruited participants from Connect (*n* = 300; M_{age} = 38; 130 females, 162 males, 6 non-binary, 3 missing) and paid them $.50. Participants were told that they would learn about a game and then answer questions about it. They were then randomly assigned to imagine playing the profitable version of the game as Player 1 (*n* = 150) or Player 2 (*n* = 150). Participants were then asked how much they trusted Player 3 on a 1-7 Likert scale ranging from “Not at all” to “Completely.” They were also asked to make the binary choice of whether Player 1 cared more about “Maximizing money” or “Fair treatment.” Lastly, they were asked to make the binary choice about whether it was more important that Player 3 eliminates money from Player 1 when they do not split the money or whether it was more important that Player 3 does not eliminate money from Player 1 when Player 1 splits the money evenly with Player 2.

To analyze trust of Player 3 between the conditions, we conducted a between-subjects ANOVA. Results revealed that participants in the Player 1 condition were significantly less likely to trust Player 3 (*M* = 2.64, *SE* = .13) compared those in the Player 2 condition (*M* = 3.19, *SE* = .13) $F(1, 298) = 9.31$, $p = .002$, $\eta^2 = .03$. A chi-square test revealed that participants in the Player 1 condition were significantly more likely to say that they cared more about being treated fairly (50%, *SE* = 4%) than maximizing the amount of money they make compared those in the Player 2 condition (31%, *SE* = 4%) $\chi^2(1, 300) = 10.83$, $p < .001$, $\varphi = -.19$. A chi-square test also revealed participants in the Player 2 condition were significantly more likely to believe that it is more important in the game for Player 3 to punish Player 1 for unfair offers than to abstain from punishing Player 1 for fair offers (33%, *SE* = 4%) compared those in the Player 1 condition (17%, *SE* = 3%) $\chi^2(1, 300) = 10.33$, $p = .002$, $\varphi = -.19$.

Experiment 8 Pre-registered: Player 1 and Player 2 Perceptions of Paid Punishment Condition

We recruited participants from Connect and paid them $.50. The judgment experiment was completely identical to the pilot.

Game Instructions

How the game works:

In the game, Player 1 is provided with $20 dollars. ***Player 1 can either keep the entire $20 for themselves, or they can keep $10 and allocate the other $10 dollars to Player 2.***

Following Player 1's decision, ***Player 3 can earn a bonus payment by eliminating some money from Player 1's final amount regardless of what Player 1 decides.*** *While Player 3 keeps the bonus payment, Player 3 does not get to keep the eliminated money for themselves, nor does the money go to Player 2; the money is simply eliminated from whatever Player 1's final amount is.*

All decisions are binding. *Once all participants have made their decisions, some participants may be randomly selected to have their games played out for actual money as part of a lottery.*

Player 1 Questions

Imagine playing the game as ***Player 1.***

As Player 1, how much do you trust Player 3?

As Player 1, do you think you would care more about maximizing how much money you make or being treated fairly by Player 3 in the game?

As Player 1, which is more important in the game - That Player 3 eliminates money from you when you keep all of the money or that Player 3 does not eliminate money from you when you split the money evenly?

Player 2 Questions

Imagine playing the game as ***Player 2.***

As Player 2, how much do you trust Player 3?

As Player 2, do you think Player 1 cares more about maximizing how much money they make or being treated fairly by Player 3 in the game?

As Player 2, which is more important in the game - That Player 3 eliminates money from Player 1 when they keep all of the money or that Player 3 does not eliminate money from Player 1 when they split the money evenly?

Experiment 9 Pilot: Perceptions of Paid and Unpaid Punishment Conditions

We recruited participants from Connect (n = 301; M_{age} = 38; 155 females, 142 males, 2 non-binary, 2 missing) and paid them $.50. Participants were told that they would learn about a game and then answer questions about it. They were then randomly assigned to imagine playing the paid (n = 150) or unpaid version of the game (n = 150). Participants were then asked how much they trusted Player 2 on a 1-7 Likert scale ranging from “Not at all” to “Completely.” They were also asked to make the binary choice of about the purpose of the game, between “The aim of players is to maximize their earning” or “The aim of players is to achieve a fair outcome”.

To analyze trust of Player 3 between the conditions, we conducted a between-subjects ANOVA. Results revealed that participants in the paid condition were significantly less likely to trust Player 3 (M = 3.30, SE = .10) compared those in the unpaid condition (M = 3.86, SE = .10), $F(1, 299) = 15.59$, $p < .001$, $\eta^2 = .05$. A chi-square test revealed that participants in the paid condition were significantly more likely to believe that the game was about maximizing earnings over achieving fair outcomes (74%, SE = 4%) those in the unpaid condition (53%, SE = 4%) $\chi^2(1, N = 300) = 14.34$, $p < .001$, $\varphi = .22$.

Experiment 9 Pre-registered: Perceptions of Paid and Unpaid Punisher Conditions

We recruited participants from Connect and paid them $.50. The judgment experiment was completely identical to the pilot.

Paid Game Instructions

How the game works:

In the game, Player 1 is provided with $20 dollars. ***Player 1 can either keep the entire $20 for themselves, or they can keep $10 and allocate the other $10 dollars to Player 2.***

Following Player 1's decision, ***Player 3 can earn a bonus payment by eliminating some money from Player 1's final amount regardless of what Player 1 decides.*** *While Player 3 keeps the bonus payment, Player 3 does not get to keep the eliminated money for themselves, nor does the money go to Player 2; the money is simply eliminated from whatever Player 1's final amount is.*

All decisions are binding. *Once participants have made their decisions, they may be randomly selected to have their game played out for actual money as part of a lottery.*

Unpaid Game Instructions

How the game works:

In the game, Player 1 is provided with $20 dollars. ***Player 1 can either keep the entire $20 for themselves, or they can keep $10 and allocate the other $10 dollars to Player 2.***

Following Player 1's decision, ***Player 3 can choose to eliminate some of the money that Player 1 would end up with regardless of what Player 1 decides.*** *Player 3 does not get to keep the eliminated money for themselves, nor does the money go to Player 2; the money is simply eliminated from whatever Player 1's final amount is.*

All decisions are binding. *Once participants have made their decisions, they may be randomly selected to have their game played out for actual money as part of a lottery.*

Questions

How trustworthy do you think Player 3 is?

What do you think is the objective of players in this game?

Supplementary Experiment 10: Public Goods Game

We recruited participants from Amazon's Mechanical Turk (*n* = 506; 270 females, 231 males, 4 non-binary, 1 missing) and paid them $.25. Participants were told that they would be playing a game involving five participants and that their decisions in the game were binding such that they would be played out for real money if selected by lottery. All participants played the game as Player 1, one of four contributors to the common pool, and were given an endowment of $10. Any amount contributed to the pool was multiplied by 1.5 and evenly redistributed among the four contributors. Player 5, the punisher did not contribute to or benefit from the pool but could remove $10 from any contributor's final payoff after seeing their decisions. This third-party punishment design was adapted from prior research from Bone and colleagues (2014). We manipulated the type of Player 5 participants were told they would play with. They were randomly assigned one of two conditions: Unpaid Player 5 who can remove $10 from any Player (*n* = 251) or Paid Player 5 can receive an unspecified bonus for removing $10 from any Player (*n* = 255). All participants were then asked to decide how much of the $10 they were allocated they wished to contribute to the common pool.

To analyze contributions between the conditions, we conducted a between-subjects ANOVA. Results revealed that participants in the paid condition contributed significantly less money to the pool (*M* = 5.55, *SE* = .20) compared those in the unpaid condition (*M* = 6.24, *SE* = .20), $F(1, 504) = 5.96$, $p = .015$, $\eta^2 = .012$ (see Supplementary Figure 4).

Game Instructions for Paid Player 5 Condition

How the game works*:*

In the game, each participant is provided with $10. You are in a group with five players: Players 1, 2, 3, 4, and 5. ***Players 1–4 each decide how much of their $10 to contribute to a common group pool. Each player can contribute any dollar amount from $0 to $10.***

Every dollar that is contributed to the group pool is multiplied by 1.5 and then evenly divided among Players 1–4, regardless of how much each person contributed. *For example, if each of the four players contributes $10, the total pool is $40. After being multiplied, the pool is $60, and each player receives $15. If you contribute $0 while the other three players each contribute $10, the total pool is $30. After being multiplied to $45, each player receives $11.25 from the pool, and because you kept your $10, your final total would be $21.25. If no one contributes, no money is multiplied and distributed. Player 5 does not contribute to the pool, and does not receive any portion of the redistributed money.*

After Players 1–4 have made their contribution decisions, ***Player 5 can earn a bonus payment***

by eliminating $10 from the final amount of any, some, or all Players, regardless of their decisions*. While Player 5 keeps the bonus payment, Player 5 does not get to keep the eliminated $10, nor does the $10 go to another player—it is simply removed from the targeted Player's final amount.*

You are Player 1*. You must decide how much (between $0 and $10) of your $10 to contribute to the group pool. Any amount you choose not to contribute will be kept by you and added to your final earnings.*

Player 5 will learn of all Players' choices and then decide whether to earn a bonus payment by removing $10 from you or any other Players.

All decisions are binding*. Once all participants have made their decisions, they may be randomly selected to have their game played out for actual money as part of a lottery.*

Game Instructions for Unpaid Player 5 Condition

How the game works*:*

In the game, each participant is provided with $10. You are in a group with five players: Players 1, 2, 3, 4, and 5. ***Players 1–4 each decide how much of their $10 to contribute to a common group pool. Each player can contribute any dollar amount from $0 to $10.***

Every dollar that is contributed to the group pool is multiplied by 1.5 and then evenly divided among Players 1–4, regardless of how much each person contributed. *For example, if each of the four players contributes $10, the total pool is $40. After being multiplied, the pool is $60, and each player receives $15. If you contribute $0 while the other three players each contribute $10, the total pool is $30. After being multiplied to $45, each player receives $11.25 from the pool, and because you kept your $10, your final total would be $21.25. If no one contributes, no money is multiplied and distributed. Player 5 does not contribute to the pool, and does not receive any portion of the redistributed money.*

After Players 1–4 have made their contribution decisions, ***Player 5 can choose to eliminate $10 from the final amount of any, some, or all Players, regardless of their decisions****. Player 5 does not get to keep the eliminated $10, nor does the $10 go to another player—it is simply removed from the targeted Player's final amount.*

You are Player 1*. You must decide how much (between $0 and $10) of your $10 to contribute to the group pool. Any amount you choose not to contribute will be kept by you and added to your final earnings.*

Player 5 will learn of all Players' choices and then decide whether to remove $10 from you or any other Players.

All decisions are binding*. Once all participants have made their decisions, they may be randomly selected to have their game played out for actual money as part of a lottery.*

Table S1.
Participant condition assignments for Experiment 2.

Conditions	Paid to Unpaid Punishers	Unpaid to Paid Punishers
$10 Punishment	188 (94 dictators, 94 punishers)	188 (94 dictators, 94 punishers)
$5 Punishment	124 (62 dictators, 62 punishers)	168 (84 dictators, 84 punishers)

Figure S1. Player 1 Cooperation Rates Between Punisher Sequence Order Conditions Across Rounds from Experiment 2. Error bars represent standard errors.

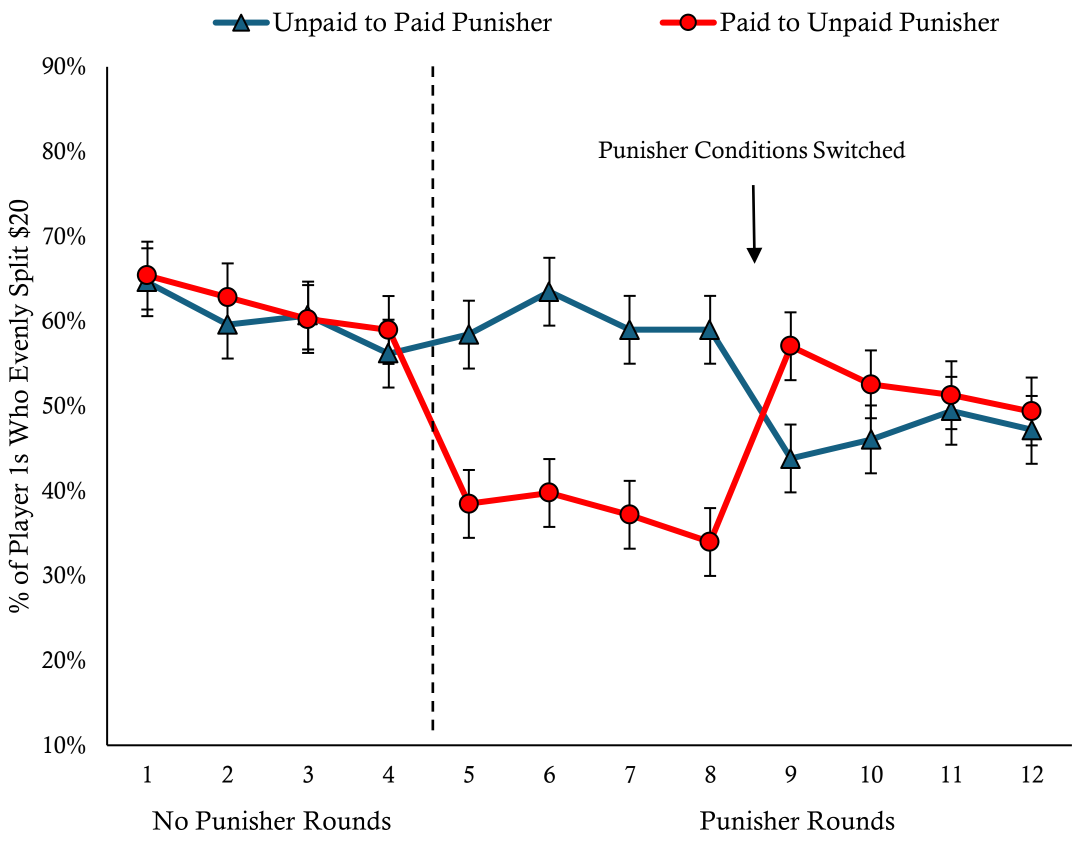

Figure S2. Player 3 Antisocial Punishment Rates Between Punisher Sequence Order Conditions Across Rounds from Experiment 2.

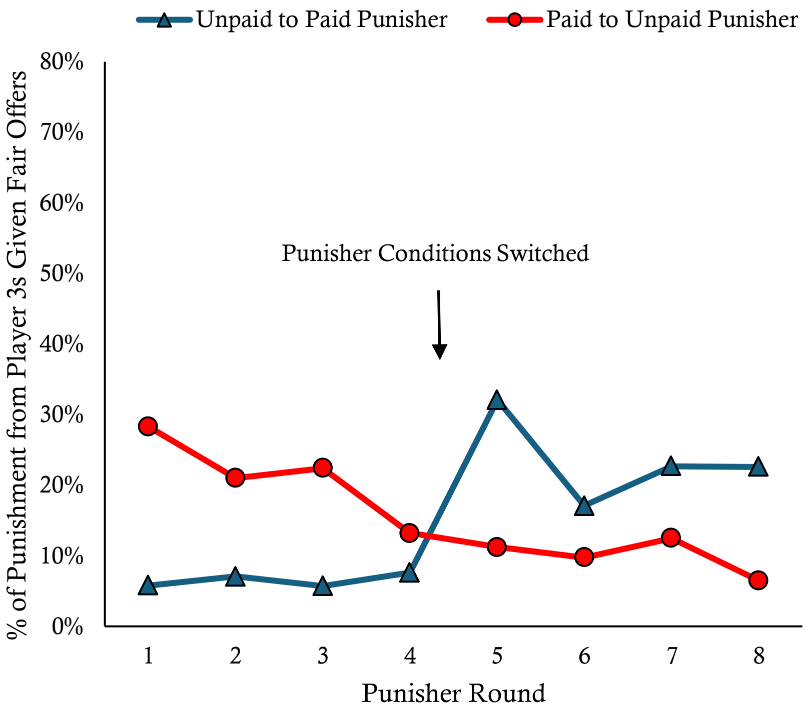

Figure S3. Player 3 Prosocial Punishment Rates Between Punisher Sequence Order Conditions Across Rounds from Experiment 2.

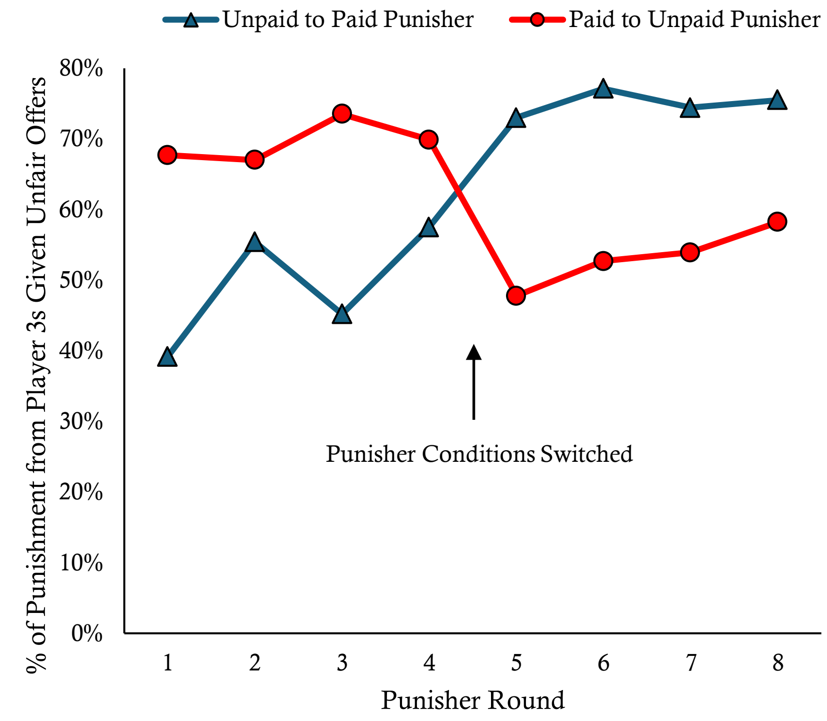

Figure S4. Average Player 1 Contribution to the Public Pool Between Punisher Conditions from Supplementary Experiment 10.

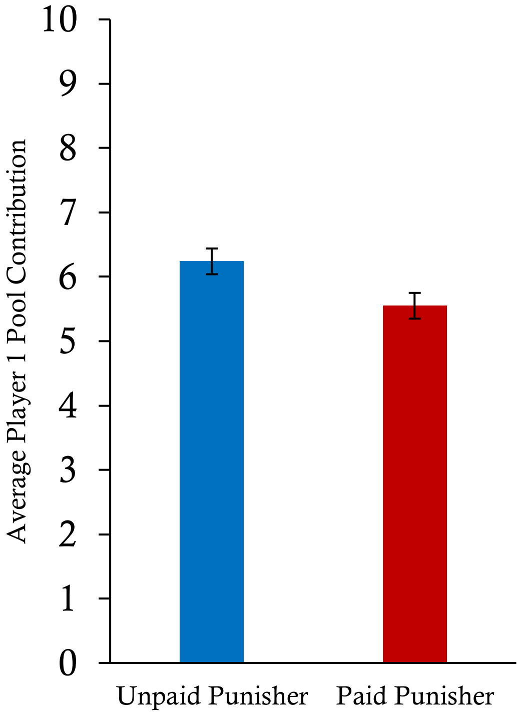

Supplementary References

Arechar, A. A., Gächter, S., & Molleman, L. (2018). Conducting interactive experiments online. *Experimental economics*, *21*, 99-131.

Bone, J., Silva, A. S., & Raihani, N. J. (2014). Defectors, not norm violators, are punished by third-parties. *Biology letters*, *10*(7), 20140388.

Molnar, A. (2019). SMARTRIQS: A simple method allowing real-time respondent interaction in Qualtrics surveys. *Journal of Behavioral and Experimental Finance*, *22*, 161-169.

Rai, T. S. (2022). Material benefits crowd out moralistic punishment. *Psychological Science*, *33*(5), 789-797.
